# Diagnosis of pupillary block glaucoma after removal of congenital cataracts with intraoperative ultrasound biomicroscopy: a case report

**DOI:** 10.1186/s12886-016-0238-9

**Published:** 2016-05-17

**Authors:** Xiang-Jia Zhu, Ke-Ke Zhang, Wen-Wen He, Xing-Huai Sun, Fan-Rong Meng, Yi Lu

**Affiliations:** Department of Ophthalmology, Eye and Ear, Nose, and Throat Hospital, Fudan University, 83 Fenyang Road, Shanghai, 200031 China; Key Laboratory of Myopia, Ministry of Health, 83 Fenyang Road, Shanghai, 200031 China; Key Laboratory of Visual Impairment and Restoration of Shanghai, Fudan University, 83 Fenyang Road, Shanghai, 200031 China

**Keywords:** Pupillary block glaucoma, Aphakic glaucoma, Ultrasound biomicroscopy, Congenital cataract, Vitreous herniation

## Abstract

**Background:**

Aphakic glaucoma is a common complication after congenital cataract extraction, especially in those who have surgery during infancy. This case report describes a case of bilateral pupillary block glaucoma diagnosed with intraoperative ultrasound biomicroscopy (UBM) after removal of congenital cataract.

**Case presentation:**

We present a case report of a 9-month-old infant with bilateral corneal enlargement and ocular hypertension after uneventful removal of congenital cataracts. Initial and follow-up examination findings were reviewed. The infant was suspected to have developmental glaucoma and schemed to have bilateral trabeculotomy until pupillary obstruction by vitreous herniation and angle closure with iris bombé were detected by intraoperative UBM. Anterior vitrectomy and goniosynechialysis were then performed as treatment.

**Conclusion:**

Pupillary block glaucoma is a rare type of infantile aphakic glaucoma. Application of intraoperative UBM can assist in the differential diagnosis of aphakic glaucoma in infants.

## Background

Pediatric glaucoma [1–4] is a vision-threatening disease that constitutes a heterogeneous group of diseases including structural abnormalities of the aqueous outflow pathways (primary glaucoma) and abnormalities affecting other regions of the eye (secondary glaucoma). Early identification and treatment of glaucoma in children is vitally important to prevent vision loss. However, identifying the exact cause of ocular hypertension can sometimes be difficult especially in children who cannot cooperate with normal ophthalmic examinations. Pediatric glaucoma occuring in aphakic eyes after removal of congenital cataracts can be even confusing.

Aphakic glaucoma is classified as secondary glaucoma and is a common complication after congenital cataract extraction, especially in those who have surgery during infancy [5, 6]. It normally develops several years after cataract surgery, although it can also occur within weeks to months of surgery [7]. The mechanism for aphakic glaucoma is unclear, as the angle is usually open on gonioscopy. Acute or subacute angle closure with iris bombé is actually a rare form of aphakic glaucoma.

Our present case shows that intraoperative ultrasound biomicroscopy (UBM) could detect angle closure with iris bombé and pupillary obstruction by vitreous herniation in an aphakic infant and help the clinician make the appropriate surgical decision.

## Case presentation

A 3-month-old female infant was admitted to our outpatient clinic on April 8, 2014 with parents’ complaints of bilateral white cloudiness at the pupillary zone perceivedd 1 month after birth. Preoperative ophthalmic examinations revealed complete bilateral lens opacification with clear corneas and anterior chambers. Since the infant did not cooperate with further examination of the posterior segment, we conducted B-scan ultrasonography of both eyes to screen abnormalities other than lens opacification, which showed negative results. Hence, she was diagnosed with bilateral congenital cataracts and underwent bilateral lensectomy, posterior capsulectomy and anterior vitrectomy under general anesthesia on April 9, 2014. Intraocular pressure (IOP) was OU 12 mmHg with bilateral corneal diameter of 10 mm measured before the corneal incision was made. Intraoperative examination and postoperative B-scan ultrasonography showed negative results of posterior segment stuctures. Therefore, the patient was discharged after an uneventful postoperative course.

**Fig. 1:**
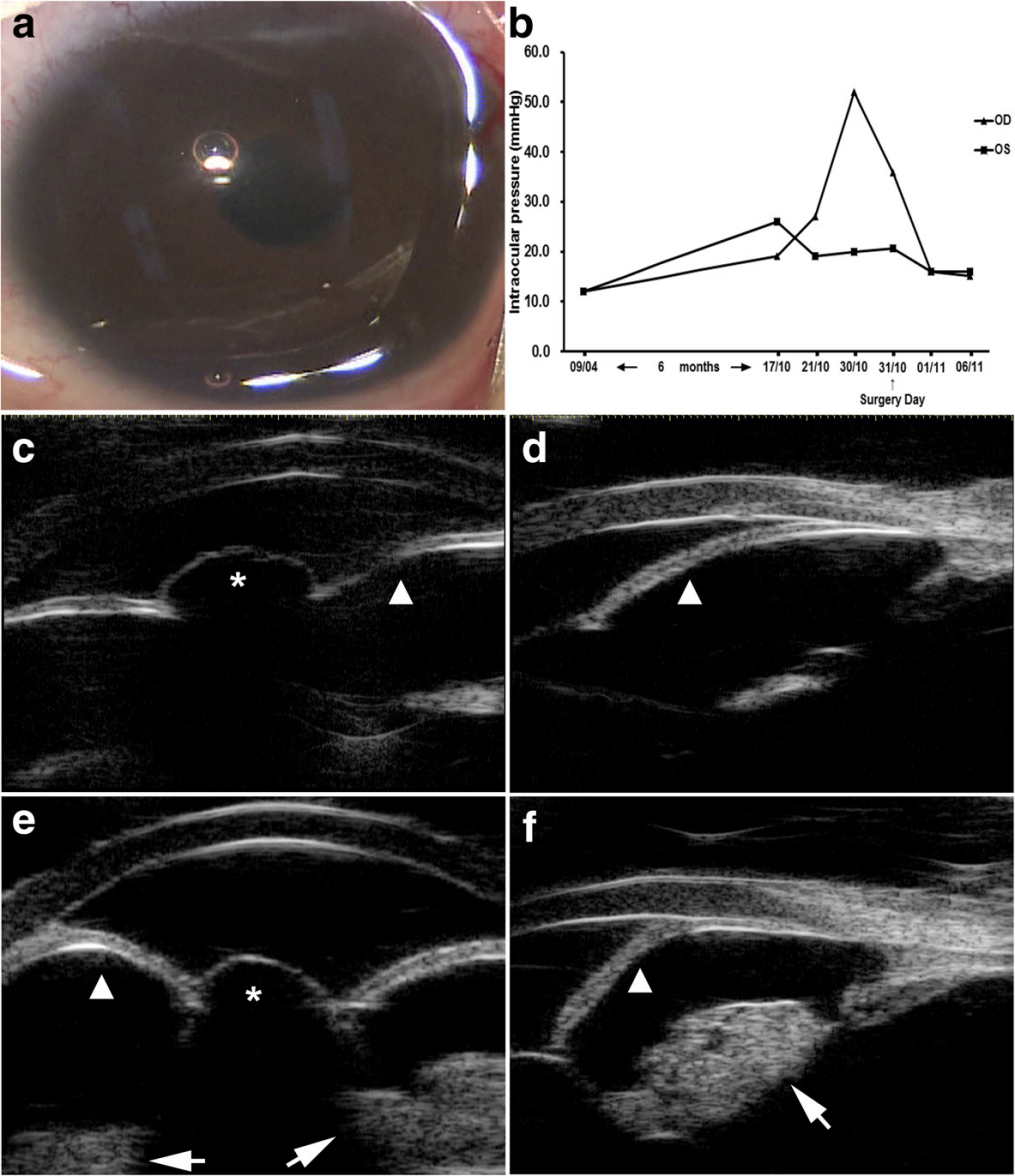
**a** Surgical video screenshots of the anterior segment of the right eye of the patient. Aphakic pupillary block glaucoma by vitreous herniation was not easily identified even under surgical ophthalmoscope. **b** The changes of bilateral intraocular pressures from April 9, 2014 to November 6, 2014. **c**-**f** Intraoperative ultrasound biomicroscopy of the right (*c and d*) and the left eye (*e and f*) captured on October 31, 2014 (prior to anterior vitrectomy and goniosynechiolysis). Absence of lens with small amount of residual cortex was detected out of the pupillary zone (*arrow*) and iris bombé (*arro*w *head*) with pupillary obstruction by vitreous herniation (*asterisk*)

However, 6 months later, the infant was suspected to have bilateral developmental glaucoma with a mild enlargement of the corneal diameter, by a local hospital. She was then referred to our hospital again. The outpatient fundus examination revealed enlarged bilateral cupping with a cup-to-disc (C/D) ratio of 0.8 OU. IOP measured by schiotz tonometer was OD 19 mmHg and OS 26 mmHg on October 17, 2014, and OD 27 mmHg and OS 19 mmHg on October 21, 2014. The patient was then admitted to our inpatient department with the tentative diagnosis of bilateral developmental glaucoma. Ophthalmic examination showed IOP OD 52 mmHg and OS 20 mmHg (October 30, 2014; schiotz tonometer), mild opacification of the superior cornea in the right eye, clear cornea of the left eye, clear bilateral anterior chamber and visual axis zone. No retinal detachment was found with B-scan ultrasonography, and A scan showed axial lengths of OD 23.43 mm and OS 20.75 mm. The patient was scheduled to receive bilateral trabeculotomy on October 31, 2014. Her intraoperative examination under general anesthesia showed IOP OD 35.8 mmHg and OS 20.6 mmHg (schiotz tonometer), superior opacification of the cornea in the right eye with a diameter of 11 mm (Fig. 1a) and a clear cornea in the left eye with a diameter of 10.5 mm, clear bilateral anterior chambers, and increased C/D ratio of OD 0.9-1.0 and OS 0.8-0.9. Gonioscopy examination showed almost overall angle closure and no detection of trabecular meshwork in both eyes, except that the superior section in the right eye was vague due to the cornea opacification. Intraoperative ultrasound biomicroscopy detected bilateral shallow anterior chambers of OD 1.75 mm and OS 1.95 mm in depth with the pupil diameter of OD 2.15 mm and OS 2.07 mm, absence of the lens with a small amount of residual cortex out of the pupillary zone, and iris bombé with pupillary obstruction by vitreous herniation in both eyes (right eye in Fig. 1c-d and left eye in Fig. 1e-f). Middle-anterior positioned iris insertion, mildly extensive and anteriorly rotated ciliary process, and 360° of peripheral anterior synechiae (PAS) and iris-trabecular meshwork contact were also observed in both eyes with UBM. Therefore, we altered the surgical plan to anterior vitrectomy and goniosynechialysis. The postoperative IOP was OU 16 mmHg. The infant was administered TobraDex Eye Drops (Alcon Laboratories, Inc., Fort Worth, TX, USA) TID OU and 1 % pilocarpine (Bausch & Lomb Freda, Shandong, China) BID OU. One week after the surgery, her IOP was OD 15 mmHg and OS 16 mmHg. The whole changes of bilateral intraocular pressures in this case was shown Fig. 1b.

During the one-year follow-up in our medical centre after surgery, the infant showed no recurrent hypertension in the right eye. The intraocular pressure in the right eye maintained between 14 to 16 mmHg using TonoPen XL tonometer (Medtronic Ophthalmics, MN, USA) after oral administration of chloralhydrate.

## Conclusions

In this case, the aphakic infant presented bilateral ocular hypertension, corneal enlargement, and increased C/D ratio 6 months after congenital cataract removal. Though no solid evidence of glaucoma was observed in the previous cataract surgery, the possibility of developmental glaucoma was not ruled out. The surgical planning was bilateral trabeculotomy with intraoperative UBM examination, which later proved to be the turning point of our treatment. The patient was identified to have pupillary block associated with vitreous herniation which eventually developed into 360 angle closure secondary to PAS. Therefore, anterior vitrectomy and goniosynechialysis was applied instead of the original surgical plan, and the surgical outcome was satisfactory.

Acute angle closure glaucoma following pediatric cataract surgery is relatively uncommon. It has been reported that the pupillary block glaucoma usually occurs soon after surgery, but the onset can also be delayed by one year or more [8]. Our case showed mild but progressive bilateral corneal enlargement and ocular hypertension occured in six months after uneventful removal of congenital cataracts. Therefore, we suggested that special caution is required when IOP elevation or corneal enlargement is noted in aphakic young children.

Furthermore, before surgical intervention and intraoperative UBM examination, several causes of ocular hypertension should be considered: 1) compromised outflow of aqueous humor by combination of abnormal development of the anterior chamber angle [9], 2) The infants’ susceptibility to inflammation induced by surgery [5], 3) the loss of lens support or vitreous factors that may obstruct the pupil, 4) infancy surgery [6, 10, 11].

Intraoperative UBM finally confirmed the diagnosis as aphakic pupillary block glaucoma in this case. In many cases, this abnormal angle configuration should be apparent under slit lamp examination, however, such a diagnosis is difficult to make in young children who cannot cooperate for normal ophthalmic examination. Aphakic pupillary block glaucoma by vitreous herniation may not be easily identified even under surgical ophthalmoscope (Fig. 1a). A recent prospective investigational study suggested that UBM is a useful device to evaluate aphakic eyes before secondary IOL implantation through good evaluation of the anterior segment and detection of any structural changes in the anterior segment resulting from the remote cause of aphakia [12]. Therefore, our case further strengthened the importance of UBM in assessing the morphology of the anterior segment in this special population.

Both IOL implantation and peripheral iridotomy may influence and/or prevent vitreous-loss pupillary block. It is reported that phacoemulsification and IOL implantation is useful in IOP control for angle closure glaucoma after relief of pupillary block [13]. Also, peripheral iridotomy could prevent recurrence of acute primary angle closure and to reduce the risk of chronic rise in IOP [14]. Some previous studies also reported that peripheral iridectomy prevented the pseudophakic pupillary block glaucoma in children [15]. In our cases, the infant will be followed up and assessed in our medical centre for the possibility of IOL implantation after two years old.

Most of the prior studies on the vitreous effect on chamber angle in aphakic patients were based more on speculation rather than solid evidence [5, 10]. In this case, with the assistance of UBM, we were able to identify those associated changes of the anterior segment. This technique greatly assisted in better understanding the cause of glaucoma in infants, especially for those with aphakic eyes after congenital cataract surgery. Intraoperative UBM examination is of substantial clinical value in differentiating the cause for elevated pressure in these patients.

### Consent

As this is a single case report, our institutional review board at Ear, Nose, and Throat Hospital of Fudan University does not require approval. The ethical standards in the Declaration of Helsinki were adhered to. Written informed consent was obtained from the parents/legal guardians of the patient for publication of this case report and any accompanying images. A copy of the written consent is available for review by the Editor of this journal.

### Availability of data and materials

All the data supporting our findings is contained within the manuscript.

## Abbreviations

C/D: cup-to-disc
IOP: intraocular pressure
PAS: peripheral anterior synechiae
UBM: intraoperative ultrasound biomicroscopy
